# Molecular Genetic Architecture of Morbid Obesity in Russian Children

**DOI:** 10.3390/biomedicines13030756

**Published:** 2025-03-20

**Authors:** Ildar R. Minniakhmetov, Rita I. Khusainova, Olga V. Vasyukova, Daria A. Kopytina, Bulat I. Yalaev, Ramil R. Salakhov, Raisat M. Guseynova, Valentina A. Peterkova, Natalia G. Mokrysheva

**Affiliations:** Endocrinology Research Centre, 117292 Moscow, Russia; khusainova.rita@endocrincentr.ru (R.I.K.); vasukova.olga@endocrincentr.ru (O.V.V.); kopytina.daria@endocrincentr.ru (D.A.K.); yalaev.bulat@endocrincentr.ru (B.I.Y.); salakhov.ramil@endocrincentr.ru (R.R.S.); guseinova.raisat@endocrincentr.ru (R.M.G.); peterkova.valentina@endocrincentr.ru (V.A.P.); mokrisheva.natalia@endocrincentr.ru (N.G.M.)

**Keywords:** obesity, whole-exome sequencing, genetic architecture of obesity

## Abstract

**Background**: Over the past few decades, the prevalence of obesity has significantly increased worldwide, particularly among children. This trend represents a global health challenge. Considering the pivotal role of obesity in the development of metabolic disorders, the identification and characterization of pathogenic gene variants in children with severe forms of obesity are key priorities in fundamental endocrinology. **Methods**: We performed whole-exome sequencing (WES) in 163 Russian children with morbid obesity and identified 96 pathogenic or likely pathogenic variants in 61 genes. These variants were clinically significant in 64 children (38.79% of the cohort). **Results**: Notably, 42 of the identified variants have not been previously described in the literature or reported in existing databases. **Conclusions**: The findings of this study will enable a more personalized approach to the diagnosis and treatment of patients with syndromic and polygenic forms of obesity. Moreover, these results advance our understanding of the genetic architecture of obesity in the Russian population.

## 1. Introduction

The World Health Organization (WHO) predicts that, by 2030, 250 million children and adolescents aged 5–19 years will be affected by obesity. This projection highlights the significance of the issue as a global health challenge with extensive consequences. A large-scale study on the prevalence of overweight among children in the Russian Federation examined 13,700 children aged 6–18 years, with a mean age of 13 years. The study identified overweight in 5.5% to 11.8% of cases and obesity in 5.5% of children living in rural areas and 8.5% of those in urban areas [1]. A study conducted in Moscow between 2017 and 2018 as part of the COSI (Childhood Obesity Surveillance Initiative) program included 2166 seven-year-old children. The study reported overweight in 27% of boys and 22% of girls, while obesity was observed in 10% and 6%, respectively [2]. DNA diagnostics is essential for understanding the underlying molecular mechanisms and improving diagnosis, prognosis, and treatment.

Advances in genomic technologies have led to the identification of numerous genetic loci associated with this condition. These studies range from severe early-onset cases and include common multifactorial polygenic forms. Methods such as whole-exome sequencing (WES) and whole-genome sequencing facilitate the identification of novel genes and biological pathways involved in syndromic and monogenic forms of obesity. However, the molecular architecture of severe childhood obesity remains poorly characterized, necessitating further investigation of causal genes. This will optimize diagnostic approaches and facilitate the development of novel therapeutic agents targeting gene products and related biochemical pathways.

Objective: The aim of this study is to identify and characterize causal gene variants in children with severe obesity using whole-exome sequencing.

## 2. Materials and Methods

This study included 163 unrelated patients diagnosed with severe early-onset obesity, who were examined at the Endocrinology Research Centre. Written informed consent was obtained from each patient or their legal representative. This study was approved by the Bioethics Committee of the Endocrinology Research Centre (protocol №18, dated 10 October 2022). A detailed description of the study cohort is presented in Table 1.

Inclusion criteria: Girls and boys with obesity onset before the age of 7 years and a BMI SDS (standard deviation score) greater than 3.5. Exclusion criteria: The presence of organic central nervous system pathology.

The study protocol included a clinical examination of patients, a detailed collection of family history, a physical examination, and an assessment of phenotypic features and anthropometric parameters. Height SDS and BMI SDS were calculated using the Auxology 1.0 software (Pfizer, USA). Laboratory diagnostics included analysis of glycated hemoglobin (HbA1c), hemoglobin, total and ionized calcium, phosphorus, fasting blood glucose, triglycerides (TG), total cholesterol, low-density lipoproteins (LDL), high-density lipoproteins (HDL), alanine aminotransferase (ALT), and aspartate aminotransferase (AST). Hormonal profiling included the measurement of insulin levels, thyroid-stimulating hormone (TSH), free thyroxine (FT4), parathyroid hormone (PTH), insulin-like growth factor 1 (IGF-1), calcitonin, and 25-hydroxyvitamin D (25-OH vitamin D). Instrumental diagnostics included ultrasound examination of the abdominal organs, kidneys, and thyroid gland, as well as X-ray imaging of the hands and wrist joints.

Food intake was recorded using a meal frequency questionnaire. A questionnaire proposed by Dykens et al. was used to assess polyphagy [3]. Subsequently, the dietitian assessed the actual nutrition according to a diary filled in by the patient’s parents for 7 days. To assess basal metabolic rate at rest, indirect calorimetry was performed in some patients (patients over 5 years old without neurological diseases). Subsequently, the diet was adjusted taking into account the individual characteristics of the patient and the results of examinations.

Genomic DNA was extracted from peripheral blood lymphocytes using the MagPure Blood DNA Kit (Magen, Guangzhou, China). Quantitative and qualitative analysis of the extracted DNA was performed using a Nanodrop 2000 spectrophotometer (Thermo Fisher Scientific, Waltham, MA, USA) and a Qubit 2.0 fluorometer (Invitrogen, Carlsbad, CA, USA) with the Qubit dsDNA HS Assay Kit. Whole-exome libraries were prepared using the KAPA HyperPlus Kit (Roche, Basel, Switzerland) according to the manufacturer’s protocol. The prepared libraries were enriched using the KAPA HyperExome Kit (Roche, Basel, Switzerland). Sequencing was performed on the Novaseq 6000 platform (Illumina, San Diego, CA, USA), using the Novaseq 6000 S4 Reagent Kit v1.5 (200 cycles) with paired-end sequencing at 2 × 100 bp. Result validation was performed using specific primers designed for the targeting of fragments of genes by Sanger sequencing on an AB3500 platform (Applied Biosystems, Waltham, MA, USA).

NGS sequencing data were processed using an automated algorithm that included alignment to the human reference genome (GRCh38), post-alignment processing, variant calling, quality filtering, and annotation of identified variants for all known transcripts of each gene from the RefSeq database. Pathogenicity prediction was performed using computational algorithms in accordance with the recommendations of the American College of Medical Genetics and Genomics (ACMG) [4].

## 3. Results

As a result of the conducted studies, 72 children (out of 163 examined) with morbid obesity (43.63%) were found to have various variants of pathogenic and likely pathogenic clinical significance. In eight patients, the identified variants were classified as ‘incidental’ findings associated with heterozygous carriage of pathogenic variants linked to the following diseases: thiamine-responsive megaloblastic anemia (OMIM: 249270)—c.1223+1G>A (*SLC19A2*), cortisone reductase deficiency type 1—c.787G>A, p.Val263Ile (*H6PD*), malignant hyperthermia—c.356G>A, p.Arg119His (*CACNA1S*), combined pituitary hormone deficiency type 3—c.1018C>T, p.Pro340Ser (*LHX3*), absorptive hypercalciuria—c.2535_2537 (rs767527947, ADCY10), Alström syndrome—c.8380C>G, p.Gln2794Glu (*ALMS1*), MEN type IV—rs755225286 (*CDKN1B*), and Wolman disease/cholesteryl ester storage disease—c.894G>A (*LIPA*).

After excluding incidental findings, the cohort with identified variants was reduced to 64 children (38.79% of the total number of examined children), in whom 96 pathogenic or likely pathogenic variants were identified in 61 genes (*ABCC8*, *ADCY3*, *ADNP*, *ADRB2*, *ALMS1*, *ANKRD17*, *ARID1B*, *ASH1L*, *AVPR2*, *BAP1*, *BBS1*, *BBS10*, *CEP290*, *CREB3L3*, *CREBBP*, *CYFIP2*, *DYRK1B*, *ENPP1*, *FAT1*, *FGFR1*, *GCK*, *GNAS*, *GPR12*, *KCNJ11*, *KCNJ15*, *KIDINS220*, *KSR2*, *LEP*, *LEPR*, *LIPE*, *MAPK8IP1*, *MC3R*, *MC4R*, *METTL5*, *MLC1*, *MYT1L*, *NAA10*, *NEXMIF*, *NTRK2*, *ODC1*, *PACS1*, *PCSK1*, *PDE11A*, *PDE4D*, *PHIP*, *PLIN1*, *POMC*, *RPL13*, *SIM1*, *SLC2A2*, *SOS1*, *SPEN*, *SPTAN1*, *STAG1*, *TNPO2*, *TRAPPC9*, *TRIP12*, *TUB*, *WDR11*, *WFS1*, *ZMYND11*, three large deletions, and one duplication). Variants in three genes—*ADCY3*, *GCK*, and *POMC*—co-occurred with autosomal dominant mutations in key genes associated with both diabetes and obesity.

**Figure 1:**
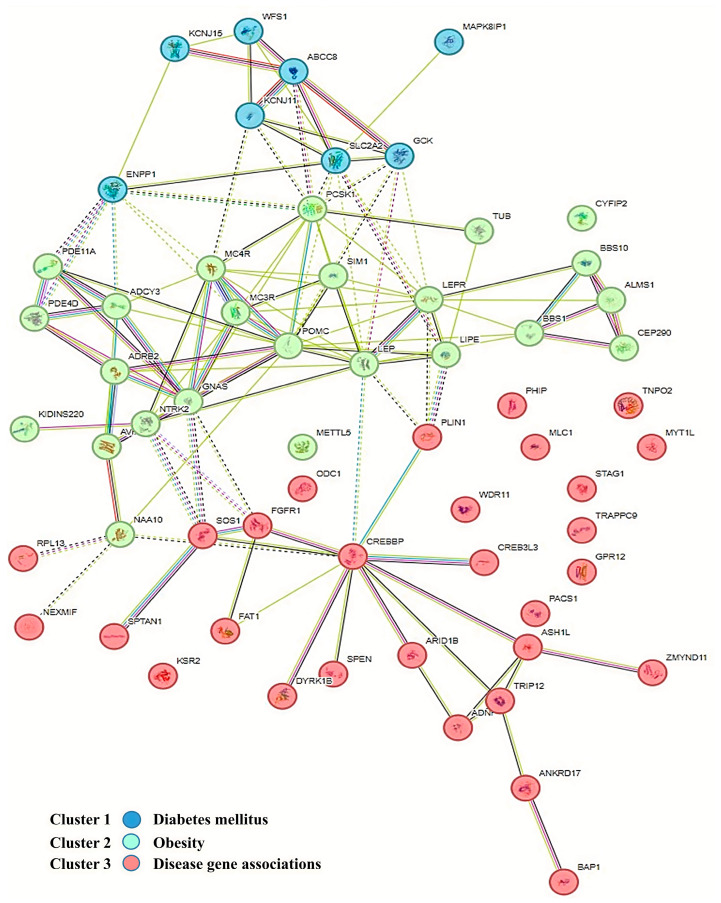
Network illustrating the clustering of protein products of genes associated with diabetes, obesity, and rare syndromes, based on genotype–phenotype correlation databases and using the k-means clustering algorithm in the STRING version 10 software.

The identified pool of genes can be categorized into three groups (Figure 1):

1. Associated with obesity;
2. Associated with diabetes and insulin resistance;
3. Associated with other comorbid conditions, typically intellectual developmental disorders.

During screening for glucose metabolism disorders in our patient cohort, normoglycemia was observed in 80% of cases, while 13% of patients exhibited impaired fasting glucose or impaired glucose tolerance. Type 2 diabetes mellitus (T2DM) was confirmed in 7% of patients. To differentiate between autoimmune and non-autoimmune forms of diabetes, specific pancreatic autoantibodies (anti-GAD, anti-insulin, ICA, and IA2 autoantibodies) were assessed in all patients. The autoantibody titers were within the reference range in all cases, suggesting a non-autoimmune etiology and supporting the diagnosis of T2DM.

Dyslipidemia was identified in 42% of cases based on national clinical guidelines. Non-alcoholic fatty liver disease (NAFLD), manifesting as hepatic steatosis, was diagnosed in 45% of patients, and 25% had steatohepatitis. The remaining 30% of patients had normal liver enzyme levels and no abnormalities were detected on ultrasound examination.

Calcium and phosphate levels, along with parathyroid hormone (PTH) and calcitonin, were assessed in patients with suspected pseudohypoparathyroidism. All patients had elevated PTH levels and increased calcitonin concentrations. In rare cases, calcium levels were within the reference range at the time of examination; however, most patients exhibited hypocalcemia and hyperphosphatemia. Additionally, patients with pseudohypoparathyroidism exhibited TSH resistance, characterized by elevated TSH levels with normal free T4 concentrations. These patients were treated with calcitriol and levothyroxine sodium.

Kidney ultrasound was performed in this patient subgroup to detect renal microcalcifications, identifying renal microliths in 75% of cases [5].

Thyroid function was also assessed in patients with other forms of obesity. Euthyroidism was observed in 92% of cases, while subclinical hypothyroidism (TSH < 10 mIU/L) was diagnosed in 8% of cases. Levothyroxine sodium therapy was not indicated for these patients due to the mild nature of thyroid dysfunction.

Serum 25-hydroxyvitamin D levels were measured in all patients. Vitamin D deficiency of varying severity was diagnosed in 87% of cases, and cholecalciferol supplementation was prescribed to restore adequate vitamin D levels.

The first group included 29 patients (43.94%), in whom 36 pathogenic/likely pathogenic variants were identified in 21 genes, as well as two large chromosomal duplications/deletions on chromosomes 17 and 15, respectively (Table 2). The second group included 7 patients (13.64%) with 11 pathogenic/likely pathogenic variants identified in 9 genes (Table 3). The third group included 28 patients (42.42%) with 36 pathogenic/likely pathogenic variants identified in 34 genes, as well as two large chromosomal deletions on chromosomes 6 and 22 (Table 4).

**Figure 2:**
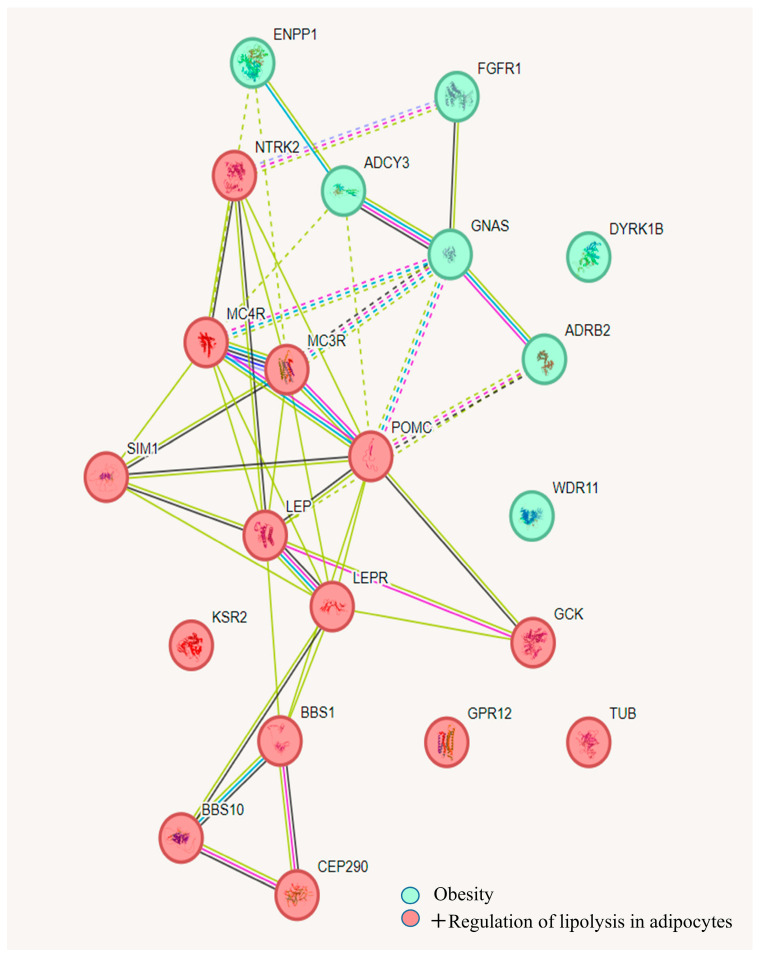
Network illustrating the clustering of protein products of genes associated with obesity and the regulation of lipolysis in adipocytes, based on gene-phenotype correlation databases and analyzed using k-means clustering algorithms in the String 10 software.

Recessive forms of severe obesity were identified, caused by homozygous or compound heterozygous mutations in the following genes: *LEP* (leptin), *LEPR* (leptin receptor), *BBS1* and *BBS10* (Bardet–Biedl syndrome), and *POMC* (pro-opiomelanocortin). These genes are well-studied, and their roles in the development of severe obesity in children are well-established. Approximately half of the identified variants had not been previously reported in the literature (Table 1). Heterozygous carriage of recessive mutations was observed in the *ADCY3* (adenylate cyclase 3), *CEP290* (centrosomal protein), and *TUB* (Tubby-like protein) genes, which are involved in obesity pathogenesis. However, no second pathogenic variant was detected. Variants were identified in the following genes: *MC3R* and *MC4R* (neuronal melanocortin receptors), *DYRK1B* (dual-specificity tyrosine-phosphorylation-regulated kinase), *GPR12* (G-protein-coupled receptor 12), *SIM1* (transcription factor), *GNAS* (stimulatory G-protein alpha subunit), *NTRK2* (neurotrophic factor receptor), and *KSR2* (kinase suppressor of Ras 2). These variants are associated with dominant forms of obesity in patients with a family history of the condition. Additionally, a previously unreported structural variation, a large deletion on chromosome 15, was identified with approximate boundaries chr15:22786711-28720475 (15q11.2-q13.1), spanning 5,933,764 base pairs. This deletion includes exons 1–5 of the *NIPA1* gene and fully encompasses the genes *MKRN3*, *MAGEL2*, imprinted genes *NDN* and *SNRPN*, and UBE3A, *GABRB3*, *GABRA5*, *OCA2*, and *HERC2*. Heterozygous deletions in the 15q11.2-q13 region with loss of the paternal copy have been described in Prader–Willi syndrome. A large duplication in a heterozygous state on chromosome 17 was also identified, with approximate boundaries chr17:6819193-7498210, spanning 679,017 base pairs. This duplication includes the genes *TEKT1*, *ALOX12P2*, *ALOX12-AS1*, *RNASEK*, *C17orf49*, *MIR497HG*, *MIR195*, *MIR497*, *BCL6B*, *SLC16A13*, *SLC16A11*, *CLEC10A*, *ASGR2*, *RPL7AP64*, *ASGR1*, *DLG4*, *ACADVL*, *MIR324*, *DVL2*, *PHF23*, *GABARAP*, *CTDNEP1*, *ELP5*, *CLDN7*, *RNA*, *SLC2A4*, *YBX2*, *EIF5A*, *GPS2*, *NEURL4*, *ACAP1*, *KCTD11*, *TMEM95*, *TNK1*, *PLSCR3*, *TMEM256*, *NLGN2*, *SPEM1*, *SPEM2*, *SPEM3*, *TMEM102*, *FGF11*, *CHRNB1*, *ZBTB4*, and *SLC35G6*. Duplications of this region with similar sizes have been described as pathogenic or likely pathogenic in the Decipher database in patients with developmental delays, obesity, short stature, and other phenotypic features. All protein products of the identified genes cluster into two subgroups: one directly involved in the development of obesity, and the other involved in lipolysis regulation in adipocytes (Figure 2).

**Figure 3:**
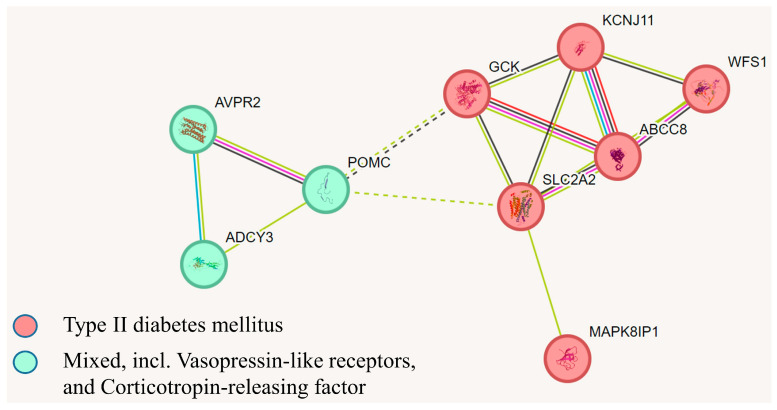
A network demonstrating the clustering of protein products of genes associated with type 2 diabetes and their interactions with vasopressin and corticotropin-releasing factors, according to gene-phenotype correlation databases, based on k-means algorithms using the String 10 software.

Pathogenic variants were also identified in genes associated with MODY (maturity-onset diabetes of the young), including *ABCC8*, *GCK*, *KCNJ11*, and *WFS1*. Variants were additionally found in genes linked to nephrogenic diabetes insipidus type 1 (*AVPR2*), the *SLC2A2* gene encoding the *GLUT2* protein, which plays a key role in glucose metabolism, and the *MAPK8IP1* gene encoding a regulator of pancreatic beta-cell function (Table 4). In one case, a mutation in the *ABCC8* gene co-occurred with a pathogenic variant in the POMC gene, while, in another case, a pathogenic variant in the *KCNJ11* gene was found alongside a mutation in the *ADCY3* gene, which encodes adenylate cyclase type 3, a protein that converts ATP to cAMP and is associated with obesity. It is known that insulin exerts a dominant anabolic effect on adipocytes by reducing the circulating concentrations of various metabolic components, stimulating glucose uptake by tissues, suppressing the release of fatty acids from adipose tissue, inhibiting ketone production in the liver, and promoting fat and glycogen storage. Accordingly, elevated insulin levels are predictably associated with weight gain [6]. Figure 3 illustrates the clustering of these genes into two subclasses: those associated with type 2 diabetes and those interacting with vasopressin and corticotropin-releasing factor. In the adenohypophysis, vasopressin, together with corticotropin-releasing hormone, stimulates the secretion of adrenocorticotropic hormone (ACTH), which is involved in stimulating lipolysis. This further suggests the involvement of the identified gene variants in the development of obesity in children.

The third group of nucleotide sequence variations in genes associated with rare syndromes is characterized as the most heterogeneous, comprising the largest number of distinct genes, primarily linked to intellectual developmental disorders (Table 4). Two cases of lipodystrophy warrant particular attention, both accompanied by various metabolic disorders, including insulin resistance, specific diabetes mellitus (lipoatrophic diabetes), hepatic steatosis, steatohepatitis, lipid metabolism disorders, and arterial hypertension. Both cases were reported for the first time.

Additionally, five patients in the third group harbored multiple molecular defects: three patients had pathogenic variants in two genes, one patient had variants in three genes, and another in four genes. This underscores the complexity of the genetic architecture of obesity associated with intellectual disabilities.

Information on the prevalence and clinical significance of the identified variants according to ACMG criteria and in silico pathogenicity assessment is presented in Table 5.

The identification of a large number of previously unreported variants emphasizes the distinctiveness of the genetic architecture of severe obesity in Russian children and highlights the need for functional studies to determine their clinical significance. This study represents the first attempt to summarize and systematize the results of whole-exome sequencing in a sufficiently large cohort of children with severe forms of obesity. Further analysis and detailed characterization of the clinical course of the disease are planned, considering the identified gene variants, family history, ethnic background, and region of residence. Additionally, dynamic monitoring of patients and their families will be performed using advanced, clinical genetic and molecular tools.

## 4. Discussion

Our study identified three groups of children with severe obesity, whose condition is likely associated with different pathogenic mechanisms. This stratification facilitates differentiation between syndromic/monogenic forms of obesity, primarily represented in the first group of children with mutations in obesity-associated genes, and the second group, where the disease likely developed due to carbohydrate metabolism disorders or complex molecular interactions of pathogenic gene variants related to lipid and carbohydrate metabolism. The third, heterogeneous group comprises children whose obesity appears to have developed due to severe disruptions in genes associated with higher nervous system development, congenital anomalies, rare syndromes, and lipid metabolism disorders. Our study identified a significant number of previously unreported variants, further increasing the value of the obtained data. Future research can focus on these variants to uncover previously unknown complex pathogenic pathways of obesity in rare syndromes or conditions that were not previously associated with severe obesity in children.

The results highlight the complexity of interpreting genetic testing results in clinical practice. Genetic obesity can be classified into two forms: syndromic, which is typically associated with chromosomal abnormalities (e.g., Prader–Willi, Fragile X, Alström, Cohen, Down syndrome, and pseudohypoparathyroidism), and non-syndromic, which includes both monogenic and polygenic obesity [7]. Monogenic obesity results from alterations in a single gene sequence or changes in chromosomal copy number due to small or large chromosomal deletions or duplications, typically inherited in a Mendelian manner. A history of early-onset obesity in children under the age of 10 serves as a basis for conducting molecular genetic testing, the choice of which depends on clinical manifestations and family history. The types of identified variants vary and include single nucleotide substitutions leading to amino acid sequence changes, substitutions resulting in protein loss of function due to frameshifts, premature stop codons, or splicing disruptions. Structural rearrangements, large deletions, or duplications affecting multiple genes, present only in the proband, can also underlie obesity [6,8]. Many of the variants identified in our study, in genes with a proven association with obesity, align with the characteristics of monogenic and syndromic forms, as well as those associated with insulin resistance disorders. On the other hand, patients with cognitive impairments represent a broad group that requires increased attention due to the established link between obesity and intellectual spectrum disorders [9].

Various forms of nonsyndromic monogenic childhood obesity exist and occur in less than 2% of children [10]. As morbid obesity is a heterogeneous condition, severe forms of the disease, including nonsyndromic early-onset forms, are likely caused by highly penetrant rare genetic variants [11]. The variants of unknown significance identified in our study, not previously reported in the literature, could potentially be associated with obesity due to a pathogenic effect. This effect could arise from high penetrance in heterozygous carriers.

Most studies have focused on the MC4R, POMC, LEP, LEPR, and BBS genes. Mutations in the melanocortin-4 receptor (*MC4R*) gene are the most common, accounting for approximately 4–6% of monogenic obesity cases. Mutations in the leptin receptor (*LEPR*) gene account for approximately 3% of cases. Mutations in other genes, such as *POMC* (pro-opiomelanocortin), *LEP* (leptin), *PCSK1* (proprotein convertase subtilisin/kexin type 1), *NTRK2* (neurotrophic receptor tyrosine kinase type 2), *BDNF* (brain-derived neurotrophic factor), and *SIM1* (single-minded family bHLH transcription factor 1), are even rarer causes of monogenic obesity [12].

In recent years, the widespread use of next-generation sequencing technologies for investigating the causes of monogenic forms of obesity has led to the identification of numerous variants of unknown clinical significance, whose relevance remains unclear [13,14]. Many authors have studied monogenic forms of obesity by analyzing a limited number of genes using custom gene panels. For example, Nordang et al. (2017) analyzed a Norwegian cohort of patients with severe obesity, including children under 12 years of age. Their gene panel included *LEP*, *LEPR*, *MC4R*, *PCSK1*, and *POMC*, and they identified four cases (0.8%) with pathogenic or likely pathogenic variants in the *MC4R* gene [15].

In a Turkish study involving 105 children with early-onset obesity and analysis of 41 genes, approximately 10.5% of the variants were found in the *SIM1*, *POMC*, *PCSK1*, *MC4R*, and *LEPR* genes [16]. Additionally, in a study of 25 patients with obesity from Guadeloupe, five heterozygous variants were identified in the *MC4R*, *NTRK2*, *SH2B1*, and *SIM1* genes, with a frequency of approximately 10% [17]. A study in Italy that screened 209 patients with obesity for mutations in the *MC4R*, *LEP*, and *LEPR* genes identified only one novel pathogenic frameshift variant in the *MC4R* gene, which disrupted gene signaling [18]. The highest prevalence of monogenic obesity was observed in a Pakistani population, where 30% of children with obesity carried pathogenic variants in the *LEP*, *LEPR*, and *MC4R* genes [19].

In 2023, Mohammed et al. conducted targeted sequencing of a panel of 52 genes (*ADCY3*, *ALMS1*, *ARL6*, *BBIP1*, *BBS1*, *BBS10*, *BBS12*, *BBS2*, *BBS4*, *BBS5*, *BBS7*, *BBS9*, *BDNF*, *CEP290*, *CFAP418*, *CPE*, *CUL4B*, *DYRK1B*, *GNAS*, *IFT172*, *IFT27*, *IFT74*, *KSR2*, *LEP*, *LEPR*, *LZTFL1*, *MAGEL2*, *MC3R*, *MC4R*, *MCHR1*, *MKKS*, *MKS1*, *MRAP2*, *NCOA1*, *NR0B2*, *NTRK2*, *PCSK1*, *PHF6*, *POMC*, *PPARG*, *RAB23*, *RAI1*, *SDCCAG8*, *SH2B1*, *SIM1*, *TMEM67*, *TRIM32*, *TTC8*, *TUB*, *UCP3*, *VPS13B*, *WDPCP*) in 243 patients with severe early-onset obesity (ages from 3 months to 10 years) observed at the Endocrinology Clinic at Sidra Medicine in Qatar. They identified 30 rare variants potentially associated with obesity in 36 of the 243 children across 15 candidate genes (*LEP*, *LEPR*, *POMC*, *MC3R*, *MC4R*, *MRAP2*, *SH2B1*, *BDNF*, *NTRK2*, *DYRK1B*, *SIM1*, *GNAS*, *ADCY3*, *RAI1*, and *BBS2*). Twenty-three of the identified variants had not been previously described in the literature at the time of this study. In this cohort, variants in the *MC4R* gene were the most common cause of obesity, with the variant c.485C>T, p.T162I being the most frequently observed mutation in five patients [20].

In Chandigarh, India, next-generation sequencing in children with obesity identified one pathogenic variant in the *MC4R* gene, one likely pathogenic variant each in the *LEPR*, *NTRK2*, and *LEP* genes, as well as variants of uncertain significance in the *POMC* and *LEPR* genes [12].

Interesting findings were reported by the group led by Petra Lloyd, who, in 2022, published results of a custom-designed panel for targeted exome sequencing in genes previously associated with copy number variations (CNVs) linked to severe childhood obesity. The authors identified 13 rare heterozygous variants of uncertain significance in 12 genes in 11 children. Two rare missense variants (p.Pro405Arg and p.Tyr232Cys) were identified in the *SORCS1* gene, which is highly expressed in the brain and has been associated with diabetes risk. Four rare variants were identified in genes within the 16p11.2 region (a frameshift variant in *TAOK2* and missense variants in *SEZ6L2*, *ALDOA*, and *KIF22*), and three rare missense variants were found in the 22q11.21 region in the *AIFM3*, *ARVCF*, and *KLHL22* genes.

Our study presents a range of novel findings, including the identification of 42 variants not previously reported in the literature. A duplication on chromosome 17 revealed two gene clusters, one of which is associated with the regulation of lipolysis in adipocytes. This cluster includes the genes *ENPP1*, *ADCY3*, *FGFR1*, *GNAS*, *ADRB2*, *DYRK1B*, and *WDR11*. Additionally, a previously unreported large deletion on chromosome 15 was identified, encompassing the genes *NIPA1*, *MKRN3*, and *MAGEL2*, the imprinted genes *NDN* and *SNRPN*, as well as *UBE3A*, *GABRB*, *GABRA5*, *OCA2*, and *HERC2*, which is of particular scientific interest. The genes *NDN*, *SNRPN*, and *UBE3A* are known for their association with Prader–Willi syndrome; *GABRB* and *GABRA5* are associated with encephalopathies; *OCA2* and *HERC2* are linked to skin pigmentation. In two patients, two distinct pathogenic variants in the *ABCC8* gene were identified, consistent with the pathogenic model of obesity development under insulin resistance induced by hyperinsulinemia. In one child, sequencing revealed two likely pathogenic variants not previously described. One of these variants, *ADCY3* c.3380C>G, is involved in the development of obesity, while the other, *KCNJ11* c.628A>G, is associated with type 2 diabetes and neonatal diabetes. A study published in *Nature Genetics* reported a direct association between pathogenic variants in the *ADCY3* gene and severe obesity in children from consanguineous Pakistani families [21], which further supports the significance of our identified variant. The combination of two previously undescribed variants located in conserved coding regions of key genes involved in energy homeostasis and receptor system regulation suggests a potential synergistic pathogenic effect. This combination may contribute to an increased risk of early-onset morbid obesity in infancy, especially given that whole-exome sequencing did not identify any other significant pathogenic variants in the patient. In one patient from the second group, nephrogenic diabetes insipidus, obesity, pronounced lentiginosis, and hypogonadotropic hypogonadism were diagnosed. Exome sequencing revealed a previously described hemizygous variant, c.262G>T, in exon 3 of the *AVPR2* gene, leading to an amino acid substitution of valine to leucine at position 88 of the protein. Vasopressin is primarily known for its role in water reabsorption through urine concentration. However, adipose tissue is also a source of metabolic water, raising the possibility that vasopressin may contribute to fat accumulation [22]. Given the impaired function of the vasopressin receptor caused by the identified pathogenic variants, disruption of these mechanisms in individuals with nephrogenic diabetes insipidus can be hypothesized. This, in part, may suggest a potential link between obesity and nephrogenic diabetes in the context of *AVPR2* gene mutations.

An intriguing finding concerns another patient from the second group, who was diagnosed with a combination of diabetes mellitus, severe obesity, optic nerve atrophy, and possibly diabetes insipidus. The patient’s brother has diabetes mellitus, obesity, and sensorineural hearing loss. Whole-exome sequencing identified two causal variants in the *WFS1* (*wolframin*) gene, found in a compound heterozygous state. One of these variants, c.1943G>A in exon 8, in a heterozygous state, has been previously described in the literature. Genome-wide association studies have identified *WFS1* variants associated with type 2 diabetes (T2D) and metabolic traits [23]. Functional studies on model animals demonstrated that mice carrying *WFS1* mutations exhibit reduced insulin response and increased cellular stress due to heightened sensitivity to a high-fat diet [24].

Heterozygous mutations in the *WFS1* gene have been associated with insulin-independent diabetes mellitus (OMIM:125853), Wolfram-like syndrome (OMIM:614296), deafness (OMIM:600965), and cataracts (OMIM:116400), following an autosomal dominant inheritance pattern. These findings strongly support the identification of causal variants in the patient, correlating with their described phenotypes.

The results of our study suggest that the pathogenesis of morbid obesity in Russian children is complex and exhibits diverse genetic architecture. The numerous variants identified in our study, many of unknown significance or probable pathogenicity, point to unexplored factors contributing to obesity.

## 5. Conclusions

Molecular genetic analysis of Russian children with morbid obesity has revealed several previously unreported genetic variants potentially associated with comorbid conditions such as obesity, insulin resistance, and syndromic forms. These findings warrant further functional studies, which may provide insights into the intricate genetic architecture of obesity. Obesity is characterized by a multifaceted metabolic imbalance that affects the regulation of energy balance, glucose homeostasis, lipid metabolism, and interactions between the central and peripheral nervous systems and adipose tissue. Many patients with diabetes and obesity meet the criteria for metabolic syndrome, which includes central obesity, dyslipidemia, and insulin resistance. However, it is often unclear which genes and causal factors contribute to the risk of obesity in such patients, as pathogenic variants are not always detected in the suspected genes. Our study brings us closer to understanding this problem. The results may serve as a guide for improving existing panels for targeted sequencing. Furthermore, previously unreported variants may help bridge the gaps in our understanding of the mechanisms underlying insulin resistance in childhood morbid obesity.

## Figures and Tables

**Table 1:** Characteristics of the study cohort.

Patient Diagnosis and Comorbidities	Number (N)	Mean Age and Standard Deviation (μ ± SD)	SDS ± SD
Total cohort	163	11.00 ± 4.00	3.95 ± 0.89
Morbid obesity	59	8.00 ± 4.56	4.03 ± 0.80
Obesity with insulin resistance or diabetes	39	13.00 ± 3.08	3.91 ± 0.73
Obesity with other comorbid conditions	65	8.00 ± 4.70	3.91 ± 0.11

Note: SDS—standard deviation score, a measure used to evaluate how closely a child’s growth matches reference data for age and sex. SD—standard deviation, a measure of how widely data points are distributed around their mean.

**Table 2:** Nucleotide sequence variations in genes associated with obesity.

Patient	Nosology Associated with the Gene	Inheritance Type	Gene, Variant	ID	Zygosity	
1	Morbid obesity due to leptin deficiency (OMIM: 614962)	AR	*LEP* (c.309C>A, p.Asn103Lys)	rs28954113	Homozygote	
2			*LEP* (c.175G>T, p.Gly59Cys)	Not reported	Heterozygote	
3	Morbid obesity (OMIM: 614963)	AR	*LEPR* (c.131_132insTGAC:p.Y44fs/c.2588_2589del:p.S863fs)	rs757358893/Not reported	Compound heterozygote	
4			*LEPR* (c.1630A>G, p.Lys544Glu/c.133_136dup, p.Tyr46Ter)	Not reported	Compound heterozygote	
5	Early-onset obesity (OMIM: 601665)	AR	*POMC* (c.616G>T, p.Glu206Ter/c.599_604dup, p.Ala201_Gln202insArgAla)	rs202127120/rs762710034	Compound heterozygote	
6	Bardet-Biedl syndrome, type 1 (OMIM: 209900)	AR	*BBS1* (c.235G>A, p.Glu79Lys/c.47+114T>C)	rs138744839/Not reported	Compound heterozygote	
7	Bardet-Biedl syndrome, type 10 (OMIM: 615987)	AR	*BBS10* (c.263T>C, p.Ile88Thr/c.145C>T, p.Arg49Trp)	Not reported/rs768933093	Compound heterozygote	
8	Obesity (OMIM: 618406)	AD	*MC4R* (c.380C>T, p.Ser127Leu)	rs13447331	Heterozygote	
9			*MC4R* (c.731C>A, p.Ala244Glu)	rs13447335	Heterozygote	
10			*MC4R* (c.202G>T, p.Ala68Ser)	rs1360504571	Heterozygote	
11	Obesity (OMIM: 618406)	AD	*MC4R* (c.731C>A, p.Ala244Glu)	rs13447335	Heterozygote	
	Non-insulin-dependent diabetes mellitus with late onset (OMIM: 125853), MODY2 (OMIM: 125851)	AD	*GCK* (c.821A>G, p.Asp274Gly)	Not reported	Heterozygote	
12	Obesity (OMIM: 618406)	AD	*MC4R* (c.750_751del, p.Ile251TrpfsTer34)	Not reported	Heterozygote	
	Hartsfield syndrome (OMIM: 615465); hypogonadotropic hypogonadism 2 with or without anosmia (OMIM: 147950); Jackson-Weiss syndrome (OMIM: 123150); osteoglophonic dysplasia (OMIM: 166250); Pfeiffer syndrome (OMIM: 101600); trigonocephaly 1 (OMIM: 190440)	AD	*FGFR1* (c.1497_1501del, p.Leu500GlnfsTer41)	Not reported	Heterozygote	
13	Obesity (OMIM: 602025)	AD	*MC3R* (c.508G>T, p.Val170Phe)	rs971796328	Heterozygote	
	Hypogonadotropic hypogonadism, 14 (OMIM: 614858)	AD	*WDR11* (c.607A>G, p.Ile203Val)	Not reported	Heterozygote	
14	Obesity (PMID: 29273807, 24209692)	AD	*KSR2* (c.1858G>A, p.Glu620Lys)	Not reported	Heterozygote	
	Obesity (OMIM: 601665); Cole disease (OMIM: 615522)	AD, AR	*ENPP1* (c.155C>T, p.Pro52Leu)	rs1408308764	Heterozygote	
15	Obesity (OMIM: 601665), Cole disease (OMIM: 615522)	AD, AR	*ENPP1* (c.2028G>T, p.Gln676His)	Not reported	Heterozygote	
16	Abdominal obesity and metabolic syndrome 3 (OMIM: 615812)	AD	*DYRK1B* (c.619C>T, p.Arg207Cys)	rs141849649	Heterozygote	
17			*DYRK1B* (c.533G>A, p.Arg178Gln)	rs1402482186	Heterozygote	
18			*DYRK1B* (c.1627C>A, p.Pro543Thr)	rs752860992	Heterozygote	
19	Dyslipidemia and obesity (PMID: 34174278, 16887097)	AD, AR	*GPR12* (c.166A>G, p.Thr56Ala)	rs1209054859	Heterozygote	
20	Severe early-onset obesity (PMID: 30991789, 28472148, 23778139, 23778136)	AD	*SIM1* (c.125del, p.Ile42LysfsTer11)	Not reported	Heterozygote	
21	Obesity and type 2 diabetes (OMIM: 109690)	Multifactorial	*ADRB2* (c.524G>A, p.Arg175Gln)	Not reported	Heterozygote	
22	Retinal dystrophy with obesity (OMIM: 616188)	AR	*TUB* (c.8G>T, p.Gly3Val)	Not reported	Heterozygote	
23	Obesity (OMIM: 617885)	AR	*ADCY3* (c.2906G>A, p.Arg969Gln)	rs371465590	Heterozygote	
24	Pseudohypoparathyroidism Ia, Ib, Ic (OMIM: 103580, 603233, 612462)	AD	*GNAS* (c.493C>T, p.Arg165Cys)	rs137854532	Heterozygote	
25		AD	*GNAS* (c.586-18_591del)	Not reported	Heterozygote	
26	Bardet–Biedl syndrome 14; Joubert syndrome 5; Meckel syndrome 4; Senior-Loken syndrome 6 (OMIM: 610142)	AR	*CEP290* (c.4267C>T, p.Arg1423Cys)	rs1353027764	Heterozygote	
27	Epileptic encephalopathy with obesity, hyperphagia, and developmental delay (OMIM: 600456)	AD	*NTRK2* (c.2143C>T, p.Arg715Trp)	Not reported	Heterozygote	
28	Developmental delay, obesity, short stature	de novo	Duplication of 17q segment, 679,017 bp	Not reported	Heterozygote	
29	Prader–Willi syndrome (OMIM: 176270)	de novo	Deletion of 15q segment, 5,933,764 bp	Not reported	Heterozygote	

**Table 3:** Nucleotide sequence variations in genes associated with diabetes mellitus and insulin resistance.

Patient	Nosology Associated with the Gene	Inheritance Type	Gene, Variant	ID	Zygosity
1	Diabetes mellitus, non-insulin-dependent (OMIM: 125853); permanent neonatal diabetes mellitus, type 3, with or without neurological features (OMIM: 618857); MODY12 (OMIM: 600509)	AD	*ABCC8* (c.3867G>A, p.Met1289Ile)	rs1467178918	Heterozygote
2	Diabetes mellitus, non-insulin-dependent (OMIM: 125853); permanent neonatal diabetes mellitus, type 3, with or without neurological features (OMIM: 618857); MODY12 (OMIM: 600509)	AD	*ABCC8* (c.560T>A, p.Val187Asp)	rs137852672	Heterozygote
	Obesity due to proopiomelanocortin deficiency (OMIM: 609734)	AR	*POMC* (c.394C>G, p.Pro132Ala)	rs8192606	Heterozygote
3	Predisposition to obesity (OMIM: 617885)	AR	*ADCY3* (c.3380C>G, p.Thr1127Ser)	rs772212679	Heterozygote
	Diabetes mellitus and familial hyperinsulinemic hypoglycemia, 2 (OMIM: 600937)	AD	*KCNJ11* (c.628A>G, p.Ile210Val)	Not reported	Heterozygote
4	Nephrogenic diabetes insipidus 1 (OMIM: 304800); nephrogenic syndrome of inappropriate antidiuresis (OMIM: 300539)	X-linked	*AVPR2* (c.262G>T, p.Val88Leu)	Not reported	Heterozygote
5	Non-insulin-dependent diabetes mellitus (OMIM: 604641)	AD	*MAPK8IP1* (c.409T>G, p.Ser137Ala)	rs1398556817	Heterozygote
	Non-insulin-dependent diabetes mellitus (OMIM: 138160)	AD	*SLC2A2* (c.1039G>A, p.Ala347Thr)	rs776435170	Heterozygote
6	MODY2 (OMIM: 125851); non-insulin-dependent diabetes mellitus with late onset (OMIM: 125853); familial hyperinsulinemic hypoglycemia, 3 (OMIM: 602485)	AD	*GCK* (c.12_13del, p.Asp4GlufsTer47)	rs1483208659	Heterozygote
7	Wolfram syndrome 1 (OMIM: 222300)	AR	*WFS1* (c.1943G>A, p.Trp648Ter/c.1244T>A, p.Val415Asp)	Not reported/rs150465110	Compound heterozygote

**Table 4:** Nucleotide sequence variations in genes associated with rare syndromes.

Patient	Syndrome Name	Gene, Variant	Inheritance Type	Description in the Literature
1	Helsmoortel–van der Aa syndrome (OMIM: 615873)	*ADNP* (c.2368G>C, p.Ala790Pro)	AD	Not reported
2	Curie–Isidor syndrome; CURI (OMIM: 619762)	*BAP1* (c.1971G>C, p.Arg657Ser)	AD	Not reported
3	Menkes–Hennekam syndrome 1; Rubinstein–Taybi syndrome 1 (OMIM: 600140)	*CREBBP* (c.3233C>T, p.Ser1078Leu)	AD	rs942848385
4	Schuurs–Hoeijmakers syndrome (OMIM: 615009)	*PACS1* (c.755C>T, p.Ser252Phe)	AD	Not reported
5	Coffin–Siris syndrome 1 (OMIM: 135900)	*ARID1B* deletion of 2734 bp	AD	Not reported
6	Chung–Jansen syndrome (OMIM: 617991)	*PHIP* (c.685T>G, p.Ser229Ala)	AD	Not reported
7	Coffin–Siris syndrome (OMIM: 135900)	Deletion of chromosome 6 segment, 2734 bp	AD	Not reported
8	Radio–Tartaglia syndrome (OMIM: 619312, RATARS)	*SPEN* (c.9354C>G, p.His3118Gln)	AD	Not reported
9	Hypertriglyceridemia, type 2 (OMIM: 619324)	*CREB3L3* (c.732dup, p.Lys245GlufsTer130)	AD	rs780374391
10	Familial partial lipodystrophy, type 6 (OMIM: 615980)	*LIPE* (c.883+1G>A/c.1419+32C>T)	AR	rs199830207, rs373163203
11	Familial partial lipodystrophy, type 4 (OMIM: 613877)	*PLIN1* (c.445G>T, p.Gly149Trp)	AD	rs200235164
12	Spastic paraplegia with intellectual disability, nystagmus, and obesity (SINO) (OMIM: 615759)	*KIDINS220* (c.5128C>T, p.Gln1710Ter)	AD	Not reported
13	Leukoencephalopathy with macrocephaly and subcortical cysts (Van der Knaap disease, OMIM: 604004)	*MLC1* (c.1027C>G, p.Gln343Glu)/deletion of chromosome 22 segment, 5331 bp	AR	Not reported
14	Syndromic microphthalmia 1 (OMIM: 309800) and Ogden syndrome (OMIM: 300855)	*NAA10* (c.20G>C, p.Arg7Thr)	X-linked	Not reported
15	Acrodysostosis, type 2, with or without hormone resistance (OMIM: 614613)	*PDE4D* (c.809-58G>C)	AD	rs1343377101
16	Primary pigmented nodular adrenal disease, type 1 (OMIM: 610475)	*PDE11A* (c.1298C>T, p.Ser433Leu)	AD	rs200330914
17	Encephalopathy, type 65, associated with developmental delay and epilepsy (OMIM: 618008)	*CYFIP2* (c.3292C>T, p.Arg1098Cys)	AD	Not reported
18	Intellectual disability with hypotonia, speech impairment, and facial dysmorphism (OMIM: 619556)	*TNPO2* (c.771+7_771+14delinsG)	AD	Not reported
19	Intellectual disability, type 13 (OMIM: 613192)	*TRAPPC9* (c.3013C>T, p.Gln1005Ter)	AR	rs913893905
20	Intellectual disability, type 30 (OMIM: 616083)	*ZMYND11* (c.1109G>A, p.Arg370Gln)	AD	rs767869912
21	Intellectual disability, type 39 (OMIM: 616521)	*MYT1L* (c.1466dup, p.Pro490AlafsTer5)	AD	Not reported
22	Intellectual disability, type 49, with macrocephaly, obesity, and tall stature (OMIM: 617752)	*TRIP12* (c.1349T>A, p.Leu450Ter)	AD	Not reported
23	Autosomal dominant intellectual disability, type 52; MRD52 (OMIM: 617796)	*ASH1L* (c.4802A>G, p.Asp1601Gly)	AD	rs373441853
24	Intellectual disability, type 72 (OMIM: 618665)	*METTL5* (c.362A>G, p.Asp121Gly)	AR	rs760916142
25	Autism spectrum disorders	*FAT1* (c.4972A>G, p.Thr1658Ala)	AD	rs754752151
		*KCNJ15* (c.1070A>T, p.Gln357Leu)	?	Not reported
26	Radio–Tartaglia syndrome (OMIM: 619312, RATARS)	*SPEN* (c.10704+2T>G)	AD	Not reported
	Endocrinopathy due to proprotein convertase 1/3 deficiency (OMIM: 600955)	*PCSK1* (c.1121G>T, p.Cys374Phe)	AR	Not reported
27	Spondyloepimetaphyseal dysplasia, Isidor-Tuten type (OMIM: 618728)	*RPL13* (c.578G>A, p.Gly193Asp)	AD	Not reported
	Chopra–Amiel–Gordon syndrome (OMIM: 619504)	*ANKRD17* (c.170T>G, p.Val57Gly)	AD	Not reported
	Developmental delay with speech impairment and behavioral abnormalities (OMIM: 619475)	*SPTBN1* (c.844G>T, p.Ala282Ser)	AD	Not reported
	Autosomal dominant intellectual disability, type 47 (OMIM: 617635)	*STAG1* (c.1562A>C, p.Gln521Pro)	AD	Not reported
28	X-linked intellectual disability, type 98 (OMIM: 300912)	*NEXMIF* (c.4313G>A, p.Gly1438Asp)	AD	Not reported
	Bachmann–Bupp syndrome (macrocephaly, alopecia, and developmental delay) (OMIM: 619075)	*ODC1* (c.508T>C, p.Phe170Leu)	AD	rs1197754776
	Noonan syndrome 4 (OMIM: 610733) and/or gingival fibromatosis 1 (OMIM: 135300)	*SOS1* (c.2566A>G, p.Ile856Val)	AD	Not reported
29	Alström syndrome (OMIM: 203800)	*ALMS1* (c.1283G>A, p.Gly428A); (c.4733T>C, p.Ile1578Thr)	AR	rs764312032, rs2103783837
	Epileptic encephalopathy 5, developmental delay with/without epilepsy, spastic paraplegia 91, distal hereditary neuronopathy 11 (OMIM: 182810)	*SPTAN1* (c.2872-2A>G)	AD	Not reported

**Table 5:** Prevalence frequencies and clinical significance of identified variants according to ACMG criteria and in silico pathogenicity assessment in predictive programs.

Gene	cDNA, Variant	Protein, Variant	ACMG Classification	Allele Frequency (gnomAD v4.1.0)	CADD Phred	In-Silico Individual Predictions (Varsome) *		
						LP/P	Uncertain	LB/B
*ABCC8*	c.3867G>A	p.Met1289Ile	VUS	6.203 × 10^7^	28.1	10	9	9
	c.560T>A	p.Val187Asp	LP	6.728 × 10^5^	27.3	6	11	5
*ADCY3*	c.2906G>A	p.Arg969Gln	VUS	9.914 × 10^6^	22.6	2	8	18
	c.3380C>G	p.Thr1127Ser	VUS	1.921 × 10^5^	22.6	2	3	30
*ADRB2*	c.524G>A	p.Arg175Gln	VUS	NA	22.9	2	8	17
*ADNP*	c.2368G>C	p.Ala790Pro	VUS	NA	26.4	16	10	0
*ALMS1*	c.1283G>A	p.Gly428A	VUS	3.718 × 10^6^	2.06	0	1	42
	c.4733T>C	p.Ile1578Thr	VUS	NA	0.02	0	1	38
*ANKRD17*	c.170T>G	p.Val57Gly	VUS	NA	24.5	5	4	16
*ASH1L*	c.4802A>G	p.Asp1601Gly	VUS	1.239 × 10^6^	26.5	0	10	14
*AVPR2*	c.262G>T	p.Val88Leu	LP	NA	24.8	17	6	0
*BAP1*	c.1971G>C	p.Arg657Ser	VUS	NA	23.1	11	5	11
*BBS1*	c.235G>A	p.Glu79Lys	VUS	0.0012	23.8	4	11	5
	c.47+114T>C	-	VUS	6.672 × 10^7^	10.3	-	-	-
*BBS10*	c.263T>C	p.Ile88Thr	VUS	NA	26.6	4	13	6
	c.145C>T	p.Arg49Trp	P	4.71 × 10^5^	31.00	6	12	2
*CEP290*	c.4267C>T	p.Arg1423Cys	VUS	6.886 × 10^6^	25.20	0	8	17
*CREB3L3*	c.732dup	p.Lys245GlufsTer130	VUS	2.782 × 10^4^	33.00	-	-	-
*CREBBP*	c.3233C>T	p.Ser1078Leu	VUS	1.487 × 10^5^	21.7	1	7	22
*CYFIP2*	c.3292C>T	p.Arg1098Cys	VUS	2.478 × 10^6^	29.8	14	3	7
*DYRK1B*	c.619C>T	p.Arg207Cys	VUS	3.037 × 10^5^	31.0	9	6	4
	c.533G>A	p.Arg178Gln	VUS	1.865 × 10^6^	24.3	6	5	14
	c.1627C>A	p.Pro543Thr	VUS	NA	19.98	0	4	28
*ENPP1*	c.155C>T	p.Pro52Leu	VUS	6.664 × 10^7^	24.3	4	5	18
	c.2028G>T	p.Gln676His	VUS	NA	3.13	2	1	39
*FAT1*	c.4972A>G	p.Thr1658Ala	VUS	2.478 × 10^6^	23.9	1	12	11
*FGFR1*	c.1497_1501del	p.Leu500GlnfsTer41	P	NA	-	-	-	-
*GCK*	c.821A>G	p.Asp274Gly	LP	NA	29.7	30	4	0
	c.12_13del	p.Asp4GlufsTer47	P	1.239 × 10^6^	26.4	-	-	-
*GNAS*	c.493C>T	p.Arg165Cys	P	NA	31.0	25	5	0
	c.586-18_591del		P	NA	-	-	-	-
*GPR12*	c.166A>G	p.Thr56Ala	VUS	1.859 × 10^6^	26.3	2	12	7
*KCNJ11*	c.628A>G	p.Ile210Val	VUS	NA	23.3	4	11	4
*KCNJ15*	c.1070A>T	p.Gln357Leu	VUS	NA	22.8	1	5	18
*KIDINS220*	c.5128C>T	p.Gln1710Ter	VUS	NA	41.0	7	2	4
*KSR2*	c.1858G>A	p.Glu620Lys	VUS	NA	22.2	2	5	18
*LEP*	c.309C>A	p.Asn103Lys	LP	4.151 × 10^5^	21.9	8	7	10
	c.175G>T	p.Gly59Cys	VUS	NA	24.32	10	10	3
	c.131_132insTGAC	p.Y44fs	LP	NA	-	-	-	-
*LEPR*	c.2588_2589del	p.S863fs	LP	NA	-	-	-	-
	c.1630A>G	p.Lys544Glu	VUS	NA	20.5	0	4	24
	c.133_136dup	p.Tyr46Ter	P	6.196 × 10^7^	17.1	-	-	-
*LIPE*	c.883+1G>A	-	LP	7.261 × 10^5^	33.0	11	3	0
	c.1419+32C>T	-	VUS/LB	6.109 × 10^5^	9.06	-	-	-
*MAPK8IP1*	c.409T>G	p.Ser137Ala	VUS	1.311 × 10^5^	13.5	0	2	44
*MC3R*	c.508G>T	p.Val170Phe	VUS/LB	NA	9.34	0	4	24
*MC4R*	c.380C>T	p.Ser127Leu	LP	0.00012	25.0	8	7	7
	c.731C>A	p.Ala244Glu	VUS	6.196 × 10^6^	25.8	21	7	0
	c.202G>T	p.Ala68Ser	VUS	6.195 × 10^7^	25.0	9	9	6
	c.750_751del	p.Ile251TrpfsTer34	P	3.284 × 10^5^	32.0	-	-	-
*METTL5*	c.362A>G	p.Asp121Gly	P	1.367 × 10^5^	32.0	7	12	3
*MLC1*	c.1027C>G	p.Gln343Glu	VUS	6.372 × 10^7^	7.92	0	4	34
*MYT1L*	c.1466dup	p.Pro490AlafsTer5	P	NA	-	-	-	-
*NAA10*	c.20G>C	p.Arg7Thr	LP	NA	23.3	2	7	17
*NEXMIF*	c.4313G>A	p.Gly1438Asp	VUS	8.256 × 10^7^	24.3	1	3	19
*NTRK2*	c.2143C>T	p.Arg715Trp	LP	NA	28.7	26	5	0
*ODC1*	c.508T>C	p.Phe170Leu	VUS/LP	1.239 × 10^6^	31.0	21	6	2
*PACS1*	c.755C>T	p.Ser252Phe	LP	NA	26.7	7	10	6
*PCSK1*	c.1121G>T	p.Cys374Phe	LP	NA	25.8	24	5	0
*PDE4D*	c.809-58G>C	-	VUS/LB	5.208 × 10^6^	15.84	-	-	-
*PDE11A*	c.1298C>T	p.Ser433Leu	VUS	5.778 × 10^5^	24.5	5	8	7
*PHIP*	c.685T>G	p.Ser229Ala	VUS	NA	26.5	5	7	14
*PLIN1*	c.445G>T	p.Gly149Trp	VUS	7.434 × 10^6^	16.52	4	4	20
*POMC*	c.616G>T	p.Glu206Ter	VUS	0.00035	39.0	2	4	2
	c.599_604dup	p.Ala201_Gln202insArgAla	VUS	0.00035	7.15	-	-	-
	c.394C>G	p.Pro132Ala	LB	0.0019	10.1	1	5	24
*RPL13*	c.578G>A	p.Gly193Asp	VUS	NA	29.4	21	6	0
*SIM1*	c.125del	p.Ile42LysfsTer11	P	NA	-	-	-	-
*SLC2A2*	c.1039G>A	p.Ala347Thr	VUS	9.131 × 10^5^	13.0	0	5	23
*SOS1*	c.2566A>G	p.Ile856Val	VUS	NA	22.2	4	9	12
*SPEN*	c.9354C>G	p.His3118Gln	VUS	NA	19.2	0	3	26
*SPEN*	c.10704+2T>G	-	LP	NA	35.0	13	2	0
*SPTAN1*	c.2872-2A>G	-	LP	NA	34.0	15	2	0
*SPTBN1*	c.844G>T	p.Ala282Ser		NA	24.5	3	11	8
*STAG1*	c.1562A>C	p.Gln521Pro	VUS	NA	27.9	9	9	6
*TNPO2*	c.771+7_771+14delinsG	-	VUS	NA	-	-	-	-
*TRAPPC9*	c.3013C>T	p.Gln1005Ter	P	6.353 × 10^7^	51.0	6	2	2
*TRIP12*	c.1349T>A	p.Leu450Ter	P	NA	39	6	2	2
*TUB*	c.8G>T	p.Gly3Val	VUS	NA	27.3	3	6	10
*WFS1*	c.1943G>A	p.Trp648Ter	P	8.05 × 10^6^	53.0	4	3	2
	c.1244T>A	p.Val415Asp	LP	NA	24.0	12	11	3
*ZMYND11*	c.1109G>A	p.Arg370Gln	VUS	5.581 × 10^6^	23.0	-	5	23

* AlphaMissense, BLOSUM, BayesDel, addAF, BayesDel, noAF, DANN, DEOGEN2, EIGEN, EIGEN-PC, EVE, FATHMM, FATHMM-MKL, FATHMM-XF, LIST-S2, LRT, M-CAP, MVP, MaxEntScan, MetaLR, MetaRNN, MetaSVM, MitImpact, MitoTip, MutPred, MutationAssessor, MutationTaster, PROVEAN, Polyphen2-HDIV, Polyphen2-HVAR, PrimateAI, REVEL, SIFT, SIFT4G, phastCons100way, vertebrate, phyloP, scSNV-ADA, scSNV-RF (https://varsome.com/about/resources/germline-implementation/#insilicopredictions) (accessed on 20 January 2025); NA—Variant not found in gnomAD, P—Pathogenic, LP—Likely Pathogenic, LB—Likely Benign, B—Benign. For CADD, variants were classified based on the fhred-like score, with a cutoff of 20; variants with scores below this threshold were considered benign, while those equal to or above 20 were classified as harmful.

## Data Availability

The original contributions presented in the study are included in the article, further inquiries can be directed to the corresponding author.

## References

[1] Vasyukova O.V. (2019). Obesity in Children and Adolescents: Diagnosis Criteria. Obes. Metab..

[2] WHO Latest Data Shows Southern European Countries Have Highest Rate of Childhood Obesity. https://dev-cms.who.int/europe/news/item/24-05-2018-latest-data-shows-southern-european-countries-have-highest-rate-of-childhood-obesity.

[3] Dykens E.M., Maxwell M.A., Pantino E., Kossler R., Roof E. (2007). Assessment of hyperphagia in Prader-Willi syndrome. Obesity.

[4] Richards S., Aziz N., Bale S., Bick D., Das S., Gastier-Foster J., Grody W.W., Hegde M., Lyon E., Spector E. (2015). Standards and Guidelines for the Interpretation of Sequence Variants: A Joint Consensus Recommendation of the American College of Medical Genetics and Genomics and the Association for Molecular Pathology. Genet. Med..

[5] Kopytina D.A., Vasyukova O.V., Salakhov R.R., Okorokov P.L., Kopytina E.V., Nagaeva E.V., Khusainova R.I., Minniakhmetov I.R., Popov S.V., Bezlepkina O.B. (2024). Identification of Novel Pathogenic Variants in the Gnas Gene in Children with Morbid Obesity and Pseudohypoparathyroidism. Obes. Metab..

[6] Ludwig D.S., Ebbeling C.B. (2018). The Carbohydrate-Insulin Model of Obesity: Beyond “Calories in, Calories Out”. JAMA Intern. Med..

[7] Singh R.K., Kumar P., Mahalingam K. (2017). Molecular Genetics of Human Obesity: A Comprehensive Review. Comptes Rendus Biol..

[8] Keller M., Svensson S.I.A., Rohde-Zimmermann K., Kovacs P., Böttcher Y. (2023). Genetics and Epigenetics in Obesity: What Do We Know so Far?. Curr. Obes. Rep..

[9] Martins L.B., Monteze N.M., Calarge C., Ferreira A.V.M., Teixeira A.L. (2019). Pathways Linking Obesity to Neuropsychiatric Disorders. Nutrition.

[10] Kelly A.S., Barlow S.E., Rao G., Inge T.H., Hayman L.L., Steinberger J., Urbina E.M., Ewing L.J., Daniels S.R. (2013). Severe Obesity in Children and Adolescents: Identification, Associated Health Risks, and Treatment Approaches: A Scientific Statement from the American Heart Association. Circulation.

[11] Serra-Juhé C., Martos-Moreno G., Bou de Pieri F., Flores R., Chowen J.A., Pérez-Jurado L.A., Argente J. (2020). Heterozygous Rare Genetic Variants in Non-Syndromic Early-Onset Obesity. Int. J. Obes..

[12] George A., Navi S., Nanda P.M., Daniel R., Anand K., Banerjee S., Panigrahi I., Dayal D. (2024). Clinical and Molecular Characterisation of Children with Monogenic Obesity: A Case Series. Pediatr. Endocrinol. Diabetes Metab..

[13] Kernohan K.D., Boycott K.M. (2024). The Expanding Diagnostic Toolbox for Rare Genetic Diseases. Nat. Rev. Genet..

[14] Roberts K.J., Chaves E., Ariza A.J., Thaker V.V., Cho C.C., Binns H.J. (2024). Exploring Genetic Testing for Rare Disorders of Obesity: Experience and Perspectives of Pediatric Weight Management Providers. Child. Obes..

[15] Nordang G.B.N., Busk Ø.L., Tveten K., Hanevik H.I., Fell A.K.M., Hjelmesæth J., Holla Ø.L., Hertel J.K. (2017). Next-Generation Sequencing of the Monogenic Obesity Genes LEP, LEPR, MC4R, PCSK1 and POMC in a Norwegian Cohort of Patients with Morbid Obesity and Normal Weight Controls. Mol. Genet. Metab..

[16] Akıncı A., Turkkahraman D., Tekedereli I., Ozer L., Evren B., Şahin I., Kalkan T., Curek Y., Camtosun E., Döğer E. (2019). Novel Mutations in Obesity-Related Genes in Turkish Children with Non-Syndromic Early Onset Severe Obesity: A Multicentre Study. J. Clin. Res. Pediatr. Endocrinol..

[17] Foucan L., Larifla L., Durand E., Rambhojan C., Armand C., Michel C.T., Billy R., Dhennin V., De Graeve F., Rabearivelo I. (2018). High Prevalence of Rare Monogenic Forms of Obesity in Obese Guadeloupean Afro-Caribbean Children. J. Clin. Endocrinol. Metab..

[18] Trevellin E., Granzotto M., Host C., Grisan F., De Stefani D., Grinzato A., Lefkimmiatis K., Pagano C., Rizzuto R., Vettor R. (2021). A Novel Loss of Function Melanocortin-4-Receptor Mutation (MC4R-F313Sfs∗29) in Morbid Obesity. J. Clin. Endocrinol. Metab..

[19] Saeed S., Bonnefond A., Manzoor J., Shabir F., Ayesha H., Philippe J., Durand E., Crouch H., Sand O., Ali M. (2015). Genetic Variants in LEP, LEPR, and MC4R Explain 30% of Severe Obesity in Children from a Consanguineous Population. Obesity.

[20] Mohammed I., Haris B., Al-Barazenji T., Vasudeva D., Tomei S., Al Azwani I., Dauleh H., Shehzad S., Chirayath S., Mohamadsalih G. (2023). Understanding the Genetics of Early-Onset Obesity in a Cohort of Children from Qatar. J. Clin. Endocrinol. Metab..

[21] Saeed S., Bonnefond A., Tamanini F., Mirza M.U., Manzoor J., Janjua Q.M., Din S.M., Gaitan J., Milochau A., Durand E. (2018). Loss-of-Function Mutations in ADCY3 Cause Monogenic Severe Obesity. Nat. Genet..

[22] Andres-Hernando A., Jensen T.J., Kuwabara M., Orlicky D.J., Cicerchi C., Li N., Roncal-Jimenez C.A., Garcia G.E., Ishimoto T., Maclean P.S. (2021). Vasopressin Mediates Fructose-Induced Metabolic Syndrome by Activating the V1b Receptor. JCI Insight.

[23] Fawcett K.A., Wheeler E., Morris A.P., Ricketts S.L., Hallmans G., Rolandsson O., Daly A., Wasson J., Permutt A., Hattersley A.T. (2010). Detailed Investigation of the Role of Common and Low-Frequency WFS1 Variants in Type 2 Diabetes Risk. Diabetes.

[24] Ivask M., Volke V., Raasmaja A., Kõks S. (2021). High-Fat Diet Associated Sensitization to Metabolic Stress in Wfs1 Heterozygous Mice. Mol. Genet. Metab..

